# Microtubule affinity regulating kinase 4 promoted activation of the NLRP3 inflammasome-mediated pyroptosis in periodontitis

**DOI:** 10.1080/20002297.2021.2015130

**Published:** 2021-12-27

**Authors:** Lulu Wang, Wenchen Pu, Chun Wang, Lang Lei, Houxuan Li

**Affiliations:** a Nanjing Stomatological Hospital, Medical School of Nanjing University, Nanjing, China; b Central Laboratory of Stomatology, Nanjing Stomatological Hospital, Medical School of Nanjing University, Nanjing, China; c Chengdu Institute of Biology, Chinese Academy of Sciences, Chengdu, China; d Laboratory of Molecular Oncology, Frontiers Science Center for Disease-related Molecular Network, State Key Laboratory of Biotherapy and Cancer Center, West China Hospital, Sichuan University, Chengdu, China

**Keywords:** MARK4, NLRP3 inflammasome, pyroptosis, periodontitis, *Porphyromonas gingivalis*

## Abstract

**Background:**

Microtubule dynamics plays a crucial role in the spatial arrangement of cell organelles and activation of the NLRP3 inflammasome.

**Purpose:**

This study aimed to explore whether microtubule affinity regulating kinase 4 (MARK4) can be a therapeutic target of periodontitis by affecting microtubule dynamics and NLRP3 inflammasome-mediated pyroptosis in macrophages.

**Materials and Methods:**

The NLRP3 inflammasome-related genes and MARK4 were measured in the healthy and inflamed human gingival tissues. Bone marrow-derived macrophages (BMDMs) were infected with Porphyromonas gingivalis, while the MARK4 inhibitors (OTSSP167 and Compound 50) and small interference RNA were utilized to restrain MARK4. Apoptosis-associated speck-like protein (ASC) speck was detected by confocal, and levels of interleukin-1β (IL-1β), as well as IL-18, were assessed by ELISA.

**Results:**

Increased staining and transcription of MARK4, NLRP3, ASC, and Caspase-1 were observed in the inflamed gingiva. *P. gingivalis* infection promoted MARK4 expression and the NLRP3 inflammasome in BMDMs. Inhibition of MARK4 decreased LDH release, IL-1β and IL-18 production, ASC speck formation, and the pyroptosis-related genes transcription. Furthermore, MARK4 inhibition reduced microtubule polymerization and acetylation in *P. gingivalis-*infected BMDMs.

**Conclusions:**

MARK4 promoted NLRP3 inflammasome activation and pyroptosis in *P. gingivalis-*infected BMDMs by affecting microtubule dynamics. MARK4 inhibition might be a potential target in regulating the NLRP3 inflammasome during periodontitis progress.

## Introduction

Periodontitis is an inflammatory disease that leads to the destruction of tooth-supporting tissues. Oral commensals contribute to microbe-host symbiosis in the periodontal niche, while excessive proliferation of *Porphyromonas gingivalis*, one keystone putative periodontal pathogen, may disrupt the periodontal homeostasis, shifting symbiosis to dysbiosis [1]. With its ample production of gingipains, outer membrane vesicles, and lipopolysaccharides (LPSs), *P. gingivalis* generates an inflammatory response that is characterized by infiltration of immune cells, such as macrophages and neutrophils.

Host immune cells are equipped with ample pattern recognition receptors on the cell membrane and in the cytosol to detect the invasion of the microbe and their products. The NLR family pyrin domain containing 3 (NLRP3) inflammasome is one important sensor of bacterial invasion in the cytosol of immune cells. After ligation with its ligand, NLRP3 recruits its adaptor, apoptosis-associated speck-like protein (ASC), to form the NLRP3-ASC complex, which sequentially cleaves pro-caspase-1 into mature caspase-1, and caspase-1 contributes to the maturation of the inflammatory cytokines interleukin-1β (IL-1β) as well as IL-18; moreover, the assembly of the NLRP3-ASC-caspase 1 complex may further cleave the Gasdermin family protein, leading to cell membrane pore formation, leakage of cellular contents and eventual occurrence of cell death, a type of regulated cell death called pyroptosis [2]. Therefore, the NLRP3 inflammasome acts as a critical sensor in the innate immune program, tightly regulating inflammatory responses and cell death [3]. The NLRP3 inflammasome plays a crucial role in the pathogenesis and development of periodontal disease [4–7]. The suppression of the NLRP3 inflammasome enables alleviation of periodontitis in a mouse model [8]; meanwhile, NLRP3 knockout reduces periodontal bone loss during *P. gingivalis* infection in mice [1].

Macrophages are the major entity of cells involved in innate immune responses to periodontal pathogens during the progress of chronic periodontitis [9,10]. They may undergo pyroptosis, apoptosis, and necroptosis (PANoptosis) in response to a low load of microbial infection [11]. By contrast, periodontal resident cells, such as epithelial cells, gingival and periodontal fibroblasts, undergo regulated cell death in response to a high load of bacterial infection [12,13]. The onset of pyroptosis in macrophages plays a crucial role in the inflammatory responses, releasing proinflammatory IL-1β and damage-associated molecular patterns (DAMPs), such as mitochondria, DNA and ribosomes, which further exacerbate local inflammation [14]. Therefore, regulating the onset of pyroptosis in macrophages may help reduce the inflammation in the periodontal microenvironment.

Microtubules, which are components of the cytoskeleton system, are crucial in the activation of the NLRP3 inflammasome by providing the platform for the assembly of the NLRP3-ASC-caspase 1 complex [15,16]. Such an assembly process requires the accumulation of acetylated α-tubulin on the microtubules to create optimal sites near the endoplasmic reticulum [17,18]. Microtubule affinity regulating kinases (MARKs), which have four family members and share a similar structure, can phosphorylate microtubule-associated proteins (MAPs) such as MAP2, MAP4 as well as tau, thereby promoting microtubule dynamics [15,19,20]. MARK4 participates in the activation of the NLRP3 inflammasome by driving it to the microtubule-organizing center, leading to the formation of one large inflammasome speck complex [21]. MARK4 has been identified as a potential drug target for several diseases such as obesity [22,23], cancer [24–26], and metabolic disorders [16].

Despite the potential role of MARK4 in the inflammasome activation, its role in periodontal diseases has never been explored. In this study, we intended to explore the role of MARK4 in regulating periodontal inflammation.

## Materials and methods

### Clinical specimens

This research was approved by the local ethics committee of the Nanjing Stomatological Hospital and Nanjing University (2016NL010 (KS)). Healthy (n = 10) and inflamed gingival tissues (n = 10) were collected as described before [16]. Healthy tissues were from sites with no attachment loss, free of bleeding on probing (BOP) and probing depths < 4 mm, and they were collected during periodontal crown lengthening, wisdom tooth extraction, or exposure of impacted teeth. Inflamed gingival tissues were taken from the free gingiva of teeth with deep periodontal pockets; they were collected during the extraction of periodontally hopeless teeth with alveolar bone resorption above 2/3, mobility up to three degrees, increased probing depths (> 6 mm), and positive BOP; and patients with periodontitis that did not undergo periodontal scaling and root planing treatment in the past six months. In addition, patients were excluded if they had: (1) systemic diseases; (2) immunodeficiency disease or immunomodulator treatment; (3) pregnancy; (4) harmful oral habits such as smoking; or (5) oral diseases.

### Cell isolation and culture

In brief, C57BL/6 mice were sacrificed by cervical dislocation and sterilized with 75% ethanol for 15 min. Then the bone marrow cells in the hind femora and tibias were flushed out and centrifugated at 1,000 rounds per min for 5 min. The erythrocytes were removed by red blood cell lysis buffer (Beyotime, China). The bone marrow cells were differentiated into murine bone marrow-derived macrophages (BMDMs) as previously described by using 1640 conditioned medium for 7 to 10 days [27]. The culture medium was composed of RPMI 1640 medium (Gibco, USA) with 20% fetal bovine serum (FBS) (Gibco, Australia), 1% penicillin/streptomycin solution, and 15% L929 conditioned medium.

### Flow cytometry

BMDMs differentiated from the bone marrow cells were identified by flow cytometry. The cells (1 × 10^5^) were stained with phycoerythrin-conjugated anti-mouse F4/80 antibody (BioLegend, 1:250, 123,109, USA) and allophycocyanin-conjugated anti-mouse CD11b antibody (BioLegend, 1:250, 101,211, USA). The stained cells were measured on FACSCalibur (BD Biosciences, USA) and analyzed with FlowJo software (Tree Star, USA).

### Bacterial culture

*P. gingivalis* (ATCC 33277) were cultured in brain heart infusion C (supplemented with 1 μg/mL yeast extract, 5 μg/mL hemin and 1 μg/mL menadione) under anaerobic conditions (85% N_2_, 5% H_2_, and 10% CO_2_) at 37 °. The BMDMs were infected with *P. gingivalis* at the exponential proliferation stage.

### Compound 50 synthesis

Compound 50 was synthesized as we described previously [28]. In brief, 2-hydroxybenzaldehyde (3 mmol), reactive methylene compound (3 mmol), and L-proline (10 mol%) were mixed and then heated under neat conditions for 0.5 h. After completion of the reaction monitored by TLC, the mixture was cooled and recrystallized from ethanol to obtain Compound 50.

### Cell viability and cytotoxicity assay

BMDMs (1 × 10^4^) were seeded in 96-well plates in 100 μL of RPMI 1640 and incubated as described before. The cells were stimulated with different concentrations of OTSSP167 (Selleck, s7159, China) or Compound 50. The viability of cells was measured by the Cell Counting Kit-8 (CCK-8) (Beyotime, China). The lactate dehydrogenase (LDH) assay (Promega, USA) was carried out according to the manufacturer’s instructions.

### Immunohistochemistry

Briefly, the healthy (n = 6) and inflamed gingival tissues (n = 6) were soaked in paraformaldehyde (4%) for 24 h and fixed with wax. Then, the tissue was cut into 3 μm thickness and incubated with anti-NLRP3 (1:100, Affinity, DF7438, China), anti-MARK4 (1:200, Proteintech, 20,174-1-AP, China), anti-ASC (1:100, Abcam, ab155970, UK), or anti-caspase-1 (1:200, CST, 2225S, China) at 4°C overnight after the regular chemical processes and followed by incubation of secondary antibodies (MaxVision, China) at 37°C for 30 min [29]. The IHC scores were determined as described previously under the chromogenic diaminobenzidine (DAKO, USA) [30]. The percentage of positive cells in ten random visual fields for healthy and periodontitis tissues, was counted with the software IHC Profiler by two independent observers who were blinded to the samples.

### Quantitative PCR

Total gingival mRNA was extracted by the QIAshredder and RNA Isolation Kit (Qiagen, Germany), while the total RNA from BMDMs was completed by a total RNA extraction reagent (Tiangen, China). The reverse transcription of RNA used the cDNA synthesis kit (Vazyme, China). The sequences of the primers for quantitative real-time PCR (qPCR) were inquired by PrimerBank (https://pga.mgh.harvard.edu/primerbank/) and synthesized by Genscript (Genscript, China). The sequences of primers were presented in Table 1. The relative quantification was calculated by the method of comparative 2^−∆∆Ct^.

**Table 1.** The sequences of primers

Gene	Genbank ID	Forward Primer (5′ to 3′)	Reverse Primer (5′ to 3′)
mus-GSDMD	NM_026960	TTCAGGCCCTACTGCCTTCT	GTTGACACATGAATAACGGGGTT
mus-Caspase-1	NM_009807	AATACAACCACTCGTACACGTC	AGCTCCAACCCTCGGAGAAA
mus-ASC	NM_023258	CTTGTCAGGGGATGAACTCAAAA	GCCATACGACTCCAGATAGTAGC
mus-IL-1β	NM_008361	GCAACTGTTCCTGAACTCAACT	ATCTTTTGGGGTCCGTCAACT
mus-IL-18	NM_008360	GACTCTTGCGTCAACTTCAAGG	CAGGCTGTCTTTTGTCAACGA
mus-MARK4	NM_172279	AGTGCAGTGGGCAGTGG	GGGGTGCTAGTGCTGTCC
mus-NLRP3	NM_145827	ATTACCCGCCCGAGAAAGG	CATGAGTGTGGCTAGATCCAAG
mus-β-actin	NM_007393	GGCTGTATTCCCCTCCATCG	CCAGTTGGTAACAATGCCATGT
Caspase-1	NM_033294	TTTCCGCAAGGTTCGATTTTCA	GGCATCTGCGCTCTACCATC
ASC	NM_145182	TGGATGCTCTGTACGGGAAG	CCAGGCTGGTGTGAAACTGAA
MARK4	NM_031417	TCACCCGCATCTCCAAC	CCCCTTCCCCTCCTGTA
NLRP3	NM_001127462	GATCTTCGCTGCGATCAACAG	CGTGCATTATCTGAACCCCAC
β-actin	NM_001101	CTACCTCATGAAGATCCTCACCGA	TTCTCCTTAATGTCACGCACGATT

### Western blot

Gingival tissues were immediately stored at −80°C after excision. The tissues were cut into pieces, lysed with the RIPA lysis buffer (Beyotime, China), and homogenized with a homogenizer. BMDMs were lysed with the RIPA. Proteins were separated by SDS-PAGE (Smart-Lifesciences, China), transferred to a PVDF membrane (Millipore, USA), and blocked with QuickBlock™ blocking buffer (Beyotime, China). Rabbit anti-β-actin (1:1,000, CST, 4970 T, China), rabbit anti-NLRP3 (1:1,000, Affinity, DF7438, China), rabbit anti-caspase-1 (1:1,000, CST, 2225S, China), rabbit anti-MARK4 (1:800, Affinity, AF0693, China), rabbit anti-pro-IL-1β (1:1,000, Affinity, DF6251, China), rabbit anti-GSDMD (1:1,000, Proteintech, 20,770-1-AP, China), rabbit anti-cleaved-GSDMD (1:1,000, Abcam, ab255603, UK), mouse anti-α-tubulin (1:50,000, Proteintech, 66,031-1-Ig, China), rabbit anti-ASC (1:1,000, Abcam, ab155970, UK), mouse anti-acet-tubulin (1:50,000, Proteintech, 66,200-1-Ig, China), mouse anti-poly-tubulin (1:50,000, Proteintech, 66,375-1-Ig, China) and secondary antibodies (1:1,000, Thermo Fisher Scientific, USA) were used. The result of the color reactions was detected by ImageQuant LAS 4000.

### Enzyme-linked immunosorbent assay

The levels of mouse IL-18 and IL-1β in culture supernatants were detected by the IL-18 (JYB, China) and IL-1β (MultiSciences, China) assay kits, respectively. The optical density (OD) was read by SpectraMax M3 (Molecular Devices, Sunnyvale, CA).

### Immunofluorescence

ASC antibody (1:200, Abcam, ab155970, UK) was used for immunofluorescence in *P. gingivalis*-treated BMDMs (MOI = 50), with or without pretreatment of OTTSP167 or Compound 50 for 2 h. Mouse acet-tubulin antibody (1:500, Proteintech, 66,200-1-Ig, China) and mouse anti-α-tubulin (1:500, Proteintech, 66,031-1-Ig, China) were used for staining the structure of microtubuli. The color reactions of images were captured on the confocal microscopy (Nikon A1, Japan).

## siRNA transfection

The siRNA targeting mouse MARK4 and negative control scramble siRNA were synthesized by RiboBio (RiboBio, China). Knockdown of MARK4 was performed by lipofectamine 3000 reagents (Thermofisher, USA) according to the manufacturer’s instructions. The efficiency of MARK4 knockdown was analyzed by Western blotting and qPCR after transfection of 72 h.

### Date statistical analysis

The data were analyzed and presented by GraphPad Prism 8 (GraphPad Software Inc, La Jolla, CA). Data were expressed as mean ± standard deviation and analyzed by Student’s t-test or nonparametric Wilcoxon test. Values of p < 0.05 were considered statistically significant. All experiments were repeated independently at least three times.

## Results

### Increased MARK4 and the NLRP3 inflammasome activation in inflamed gingival tissues

**Figure 1.**
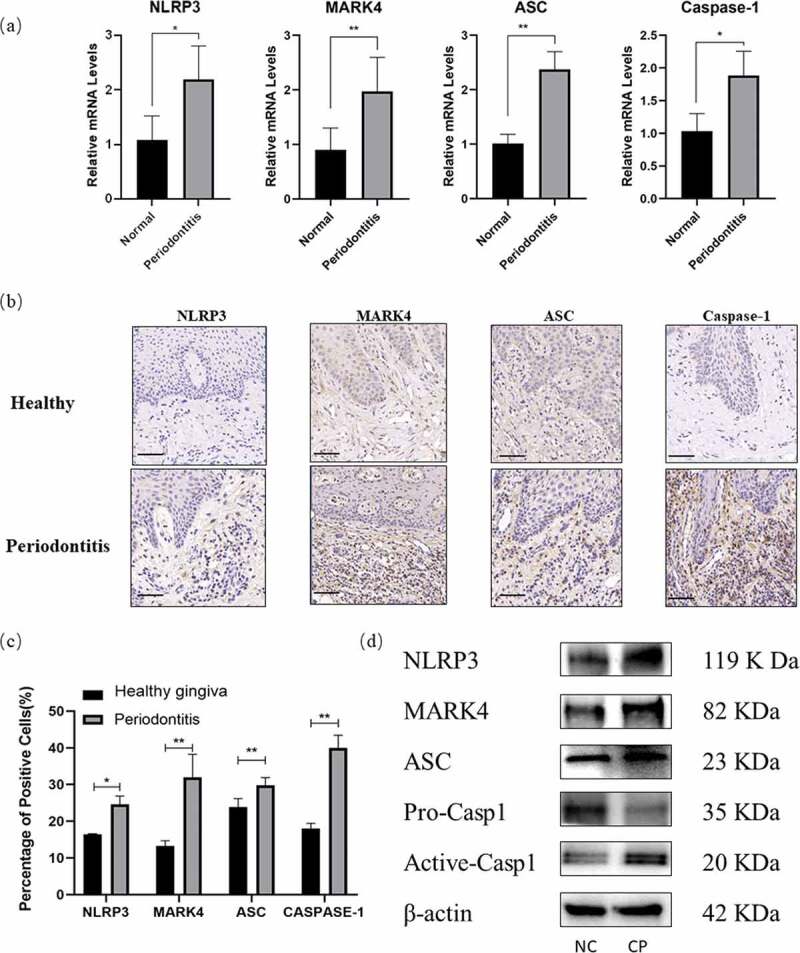
Increased MARK4 and the NLRP3 inflammasome activation in inflamed gingival tissues

First, we explored the expression of MARK4 and NLRP3 inflammasome-related genes in the gingival tissues. Levels of MARK4, NLRP3, ASC, and caspase-1 mRNA were significantly increased in the infected groups (Figure 1(a)). In addition, we examined the protein levels of MARK4 and pyroptosis-associated markers in the healthy and inflamed tissues by immunohistochemistry (Figure 1(b,c)) and Western blot (Figure 1(d)) respectively. More positive staining of MARK4, NLRP3, ASC, and caspase-1 could be detected in the inflamed than in the healthy gingival tissues. These results demonstrated that the expression of MARK4 and the NLRP3 inflammasome were upregulated in periodontitis.

(**a**) Gene transcription in the healthy (n = 10) and inflamed (n = 10) gingiva was measured by real time qPCR. (**b** and **c**) Samples from healthy (n = 6) and inflamed gingiva (n = 6) were stained by the immunolohistological chemistry. The scale bar represents 50 µm. (**d**) Protein levels in the gingival tissue from chronic periodontitis (CP, n = 3) or normal control (NC, n = 3) were evaluated by Western blot. *p < 0.05; **p < 0.01; ***p < 0.001.

### *Increased MARK4 expression and pyroptosis in* P. gingivalis*-infected BMDMs*

**Figure 2.**
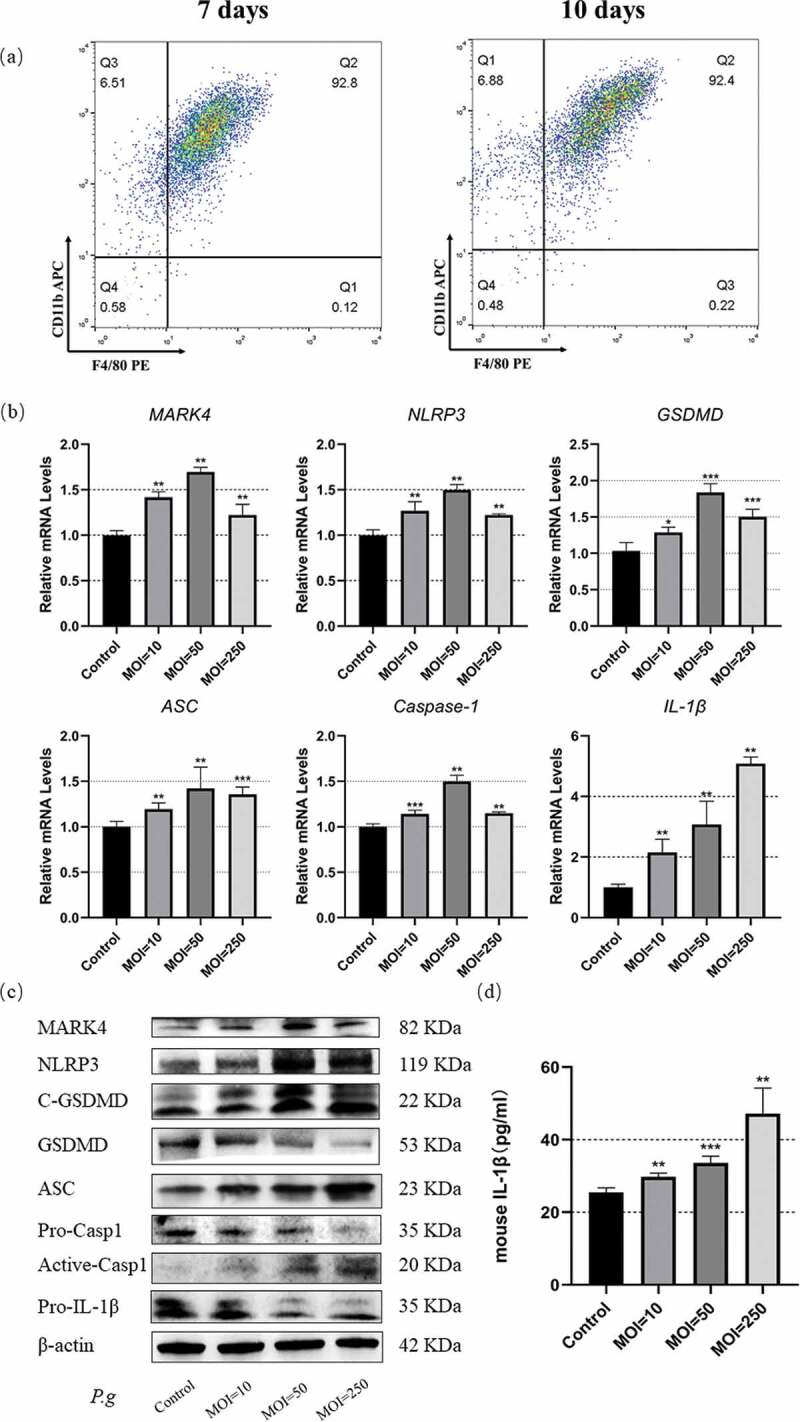
Upregulated MARK4 expression and pyroptosis in the *P. gingivalis*-infected BMDMs

To further characterize the mechanism of enhanced expression of MARK4 and pyroptosis-related genes in the inflamed periodontal tissues, we utilized *P. gingivalis* to infect BMDMs to mimic the periodontal inflammatory niche. First, we examined the expression of macrophage markers by flow cytometry, showing that CD11b^+^ F4/80^+^ cells accounted for over 90% of cultured cells after induction for 7 or 10 days (Figure 2(a)); therefore, we characterized the cultured cells as macrophages. *P. gingivalis* infection promoted the mRNA transcription of NLRP3, MARK4, GSDMD, ASC, caspase-1 and IL-1β in the BMDMs, with the highest transcription of NLRP3, MARK4, GSDMD, ASC, and caspase-1 at an MOI of 50 and IL-1β at an MOI of 250 (Figure 2(b)); meanwhile, protein levels of NLRP3, MARK4, cleaved-GSDMD, ASC, and caspase-1 were increased in *P. gingivalis-*infected BMDMs (MOI = 10, 50, and 250), whereas the expression of GSDMD, pro-caspase-1, pro-IL-1β was decreased (Figure 2(c)), suggesting the cleavage of GSDMD and caspase-1. In addition, IL-1β in the supernatants of bacteria-infected BMDMs was augmented in a time-dependent manner (Figure 2(d)). Therefore, *P. gingivalis* infection can boost the NLRP3 inflammasome activation and MARK4 activation in the BMDMs.

(**a**) BMDMs induced by L929 conditional medium for 7 days or 10 days were identified by flow cytometry. Mature BMDMs were defined as CD11b^+^F4/80^+^ cultures. (**b**) BMDMs were infected with *P. gingivalis* (MOI = 10, 50 or 250) for 2 h, and gene transcription levels were assessed by real-time qPCR (n = 3). (**c**) Protein expression was analyzed by Western blot after bacterial infection for 24 h (MOI = 50) (n = 3). (**d**) IL-Iβ in the culture supernatant was detected by ELISA. *p < 0.05; **p < 0.01; ***p < 0.001 compared with the control group.

### *Reduced pyroptosis after MARK4 inhibition in* P. gingivalis*-infected BMDMs*

**Figure 3.**
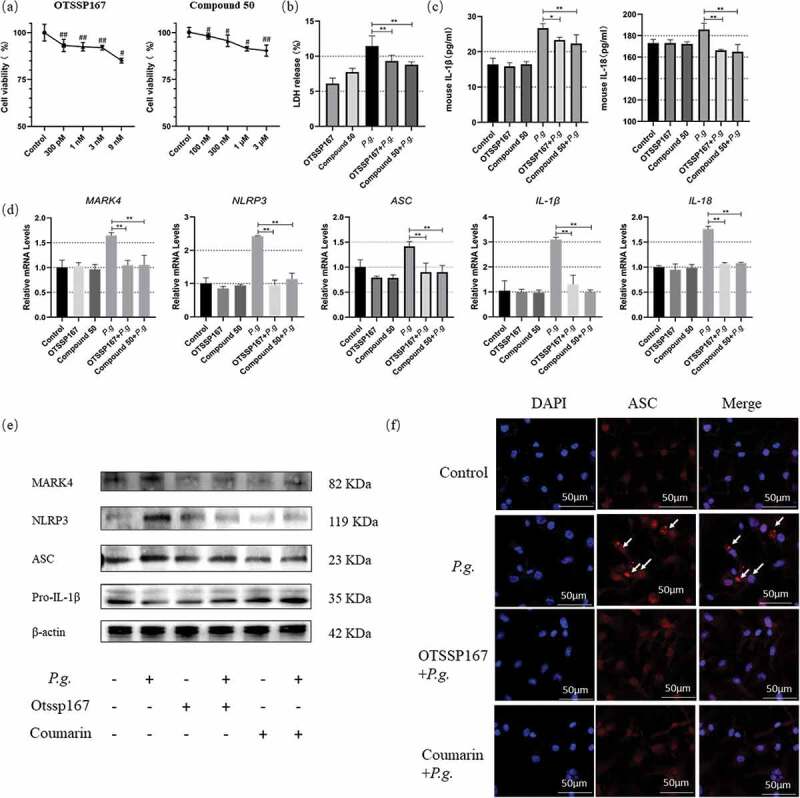
Reduced pyroptosis in *P. gingivalis*-infected BMDMs by MARK4 inhibition

To further address the relations between MARK4 and the NLRP3 inflammasome, we utilized the inhibitors of MARK4 (OTSSP167 or Compound 50) to pretreat the BMDMs before *P. gingivalis* stimulation (MOI = 50). We first assessed the cytotoxicity of the inhibitors by the CCK8 assay. Cell viability of BMDMs was more than 90% when the concentration of OTSSP167 was below 1 nM, while the cell death rate was less than 10% when the concentration of Compound 50 was below 1 μM (Figure 3(a)). OTSSP167 and Compound 50 pretreatment reduced the cell death in *P. gingivalis-*infected BMDMs (Figure 3(b)). In addition, OTSSP167 and Compound 50 suppressed the level of IL-1β and IL-18 in the supernatants of *P. gingivalis*-infected BMDMs (Figure 3(c)). Furthermore, OTSSP167 and Compound 50 pretreatment inhibited mRNA transcription of NLRP3, MARK4, ASC, IL-1β, and IL-18 (Figure 3(d)), and reduced protein levels of MARK4, NLRP3, and ASC in *P. gingivalis-*infected BMDMs (Figure 3(e)). Moreover, the MARK4 inhibitors could restrain the maturation of pro-IL-1β (Figure 3(e)). Since ASC speck would emerge near the nucleus under the assembly of the NLRP3 inflammasome, we also explored the formation of ASC speck by confocal microscopy. Inhibition of MARK4 apparently mitigated the formation of ASC speck in BMDMs infected by *P. gingivalis* (Figure 3(f)). These results collectively validated that MARK4 participated in the regulation of the NLRP3 inflammasome and pyroptosis in BMDMs.

(**a**) The cell viability was measured by the CCK8 assay. (**b**) BMDMs were pretreated with OTSSP167 (1 nM) or Compound 50 (1 μM) for 2 h and stimulated with *P. gingivalis* (MOI = 50) for 24 h, and the percentage of the death cell was measured by the LDH assay. (**c**) IL-Iβ and IL-18 in the culture supernatants of 24 h were detected by ELISA. (**d**) Gene transcription was assessed by real-time qPCR at 2 h. (**e**) Protein levels were analyzed by Western blot at 24 h. (f) The formation of ASC speck at 24 h were captured under a fluorescence microscope. White arrows indicated ASC specks. Scale bars, 50 µm. #p < 0.05; ##p < 0.01 compared with the control; *p < 0.05; **p < 0.01 compared with the *P. gingivalis*-treated group.

### *Decreased pyroptosis after MARK4 knockdown in* P. gingivalis*-infected BMDMs*

**Figure 4.**
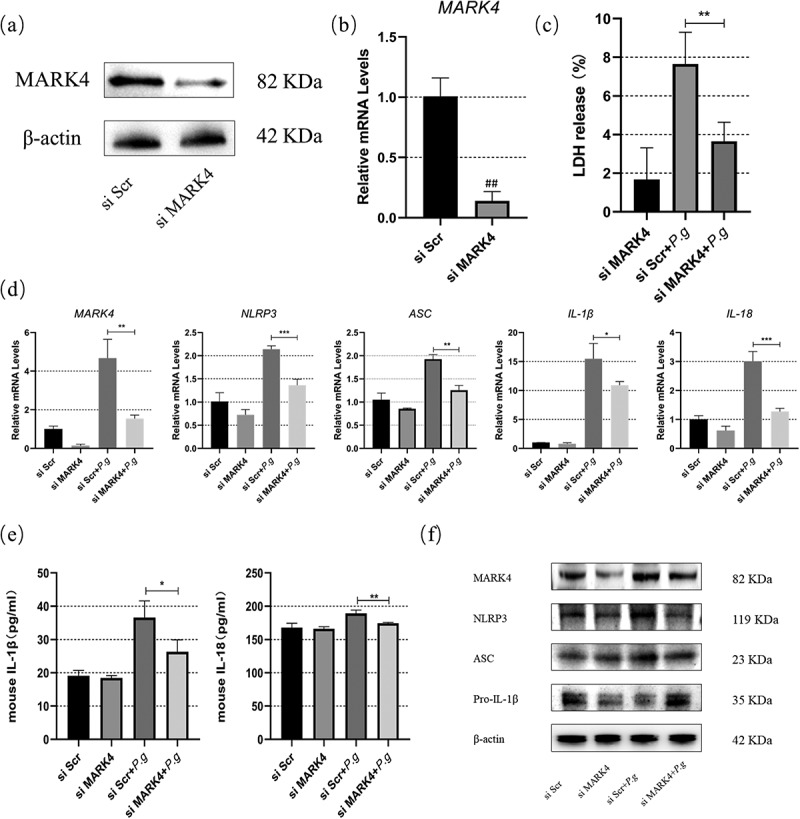
MARK4 knockdown alleviated the NLRP3 inflammasome-mediated pyroptosis in BMDMs

To further verify the regulatory role of MARK4 in the NLRP3 inflammasome, we transfected BMDMs with MARK4 siRNA before *P. gingivalis* infection. Significant knockdown of MARK4 was detected by qPCR and Western blot, respectively (Figure 4(a,b)). Cell death induced by *P. gingivalis* was diminished after MARK4 knockdown (Figure 4(c)). In addition, MARK4 knockdown inhibited the mRNA transcription of NLRP3, MARK4, ASC, IL-1β, and IL-18 in *P. gingivalis-*treated BMDMs (Figure 4(d)). Moreover, protein levels of IL-1β and IL-18 in the supernatants (Figure 4(e)) and the levels of NLRP3, MARK4, and ASC in the cell lysis were decreased after MARK4 knockdown in *P. gingivalis-*infected BMDMs (Figure 4(f)). Furthermore, MARK4 knockdown suppressed the cleavage of pro-IL-1β (Figure 4(f)). Taken together, MARK4 knockdown diminished NLRP3-mediated pyroptosis in *P. gingivalis*-treated macrophages

BMDMs were transfected with siRNA for 72 h. (**a** and **b**)The efficiency of MARK4 knockdown was detected by Western blot and qPCR, respectively. (**c**) Cell death was assessed by LDH assay. (**d**) Gene transcription in *P. gingivalis* (MOI = 50)-infected BMDMs were assessed by qPCR at 2 h. (**e** and **f**) Protein levels in the supernatants or cell lysis were detected by ELISA or Western blot at 24 h, respectively. #p < 0.05; ##p < 0.01 vs si Scr group. *p < 0.05, **p < 0.01, vs bacteria-treated siScr group.

### *MARK4 affected microtubule acetylation and polymerization in* P. gingivalis*-infected BMDMs*

**Figure 5.**
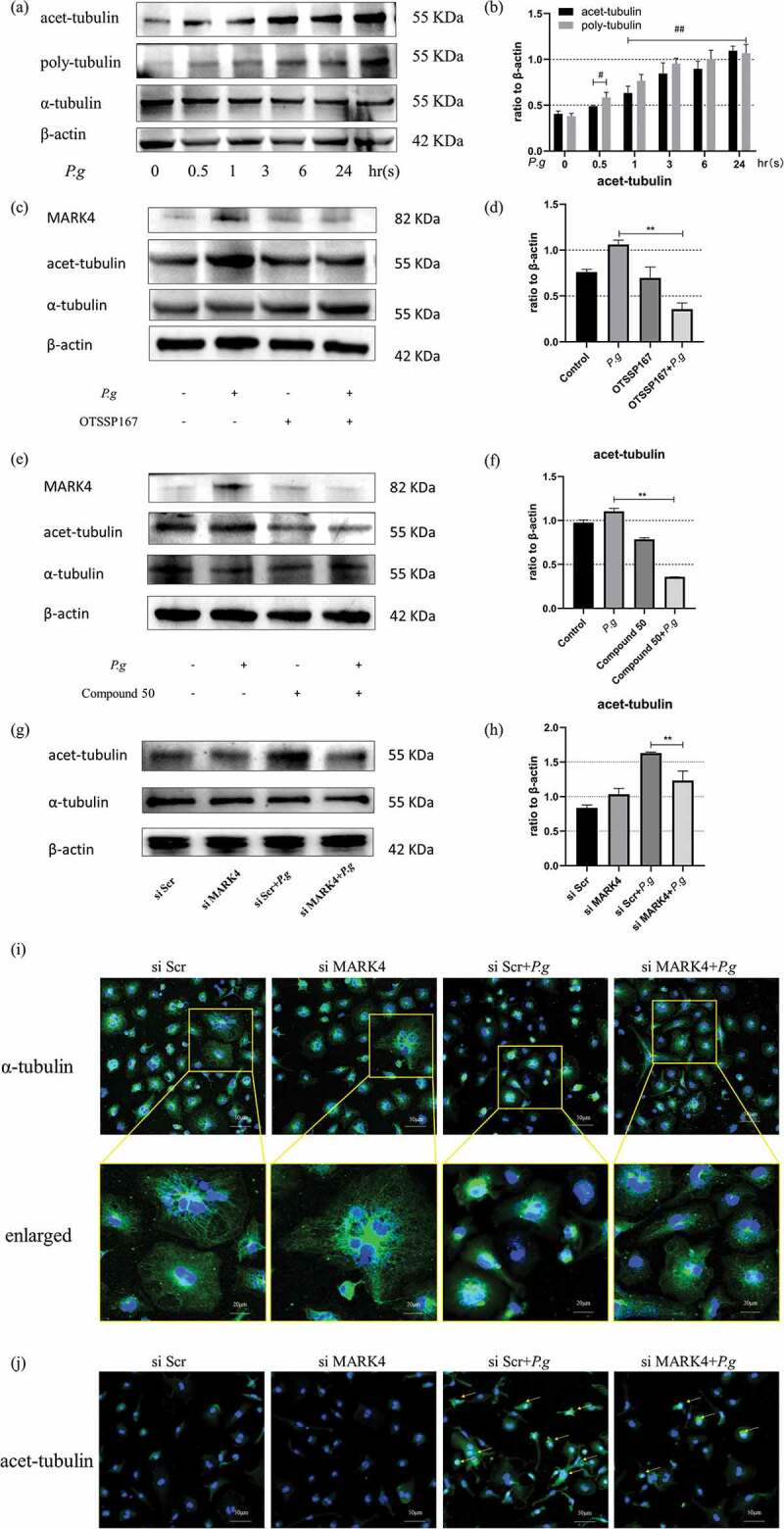
MARK4 affected microtubule acetylation and polymerization in *P. gingivalis*-infected BMDMs

The microtubule network is important for NLRP3 activation by driving the spatial arrangement of mitochondria [17]. Microtubule polymerization and acetylation are of great importance in regulating microtubule dynamics and fundamentally affects the inflammasome activation and pyroptosis [18]. To determine whether *P. gingivalis* induced changes in the microtubule dynamics in BMDMs, we infected BMDMs with *P. gingivalis* (MOI = 50) at different indicated times. Significant microtubule acetylation could be observed as early as 0.5 h with a time-dependent increase till 24 h; similarly, an increase in the polymerized tubulin could be observed at 0.5 h and kept an upward tendency as late as 24 h (Figure 5(a,b)). The increase in the tubulin acetylation was significantly reduced after OTSSP167 (Figure 5(c,d)) and Compound 50 pretreatment (Figure 5(e,f)) in *P. gingivalis*-infected BMDMs (P < 0.05). In addition, MARK4 knockdown diminished the acetylation induced by *P. gingivalis* (MOI = 50) for 24 h (Figure 5(g,h)). These findings were further confirmed by immunofluorescence staining of α-tubulin (Figure 5(i)) and acet-tubulin (Figure 5(j)). *P. gingivalis* induced microtubule polymerization in BMDMs but MARK4 knockdown preserved the microtubule network against *P. gingivalis* infection (Figure 5(i)). Moreover, knockdown of MARK4 apparently mitigated the acetylation of microtubule in BMDMs infected by *P. gingivalis* (Figure 5(j)). Therefore, MARK4 may modulate the NLRP3 inflammasome and pyroptosis by influencing the polymerization and acetylation of microtubules.

(**a** and **b**) Expression of acet-α-tubulin and poly-tubulin in BMDMs after *P. gingivalis* infection (MOI = 50) at indicated times. (**c-h**) The acetylation of microtubules at 24 h after MARK4 inhitition by OTSSP167 or Compound 50 and knockdown by siRNA. (**i**) Immunofluorescent confocal photographs of the microtubule structure (scale bars = 50 µm) and enlarged representative views (scale bars = 20 µm). (**j**) Acetylation of microtubules detected by confocal imaging after MARK4 knockdown in *P. gingivalis*-infected BMDMs. Yellow arrows indicate acetylated microtubules (**s**cale bars = 50 µm). #, p < 0.05; ##, p < 0.01 compared with the *P.g*-untreated group. *, p < 0.05, **, p < 0.01 compared with the *P.gingvitis*-treated group.

## Discussion

Host-microbiome interactions impact both innate and adaptive immune homeostasis, and such interactions play a crucial role in the progress of periodontitis [31]. It has been shown that *P. gingivalis* can induce NLRP3 inflammasome activation and promote pyroptosis in a caspase-1-dependent and -independent pathway [32,33]. Microtubules mediate the intracellular transport of various cargos, including organelles and microbials, and the spatial arrangement of mitochondria by microtubule affects the assembly of NLRP3-ASC [17]. Our present study showed for the first time that *P. gingivalis* affected the microtubule platform in the BMDMs, and MARK4 regulated the assembly of NLRP3-ASC, the release of pro-inflammatory IL-1β and IL-18, and the onset of pyroptic cell death, and such effects may be related to its effect on microtubule polymerization and acetylation. MARK4 may become a therapeutic target during the progress of periodontitis by regulating microtubule dynamics and NLRP3 inflammasome activation.

The α- and β-tubulin heterodimer, which build up the microtubules, undergoe consistent post-translational modifications (PTMs) to adapt to the changing microenvironment. The possible role of the cytoskeleton in the periodontitis progress has been noted as early as 1990 [34]. A soluble sonic extract (SSE) from *Bacteroides* (currently *Porphyromonas*) *gingivalis* inhibited the growth of gingival fibroblast, and disorganized actin stress fibers, while the microtubular network remained intact [34]. Gingipain from *P. gingivalis* promoted depolymerization of the F-actin cytoskeleton, induced cell morphological alteration, and enhanced apoptosis in osteoblasts [35]. Moreover, *P. gingivalis* strains 33,277 and W50 at MOI of 100 induced β-actin cleavage as early as 1 h leading to cytoskeleton collapse [36]. However, the role of the microtubule in the periodontitis progress has never been reported. Similar to LPS-induced polymerization of microfilaments and microtubules in monocytes and macrophages [37], *P. gingivalis* promoted microtubule polymerization and acetylation in BMDMs in our present study, and such alterations in the microtubule dynamics will fundamentally affect the inflammatory process during periodontal pathogen infection in immune cells.

Integrity of the microtubule is crucial in mediating the inflammation and NLRP3 inflammasome activation in the macrophages [17,38]. Colchicine or nocodazole, inhibitors of tubulin polymerizations, disrupt the microtubule network and suppress the activation of the NLRP3 inflammasome [21]. Microtubule acetylation is regarded as a significant mediator of the inflammasome complex assembly, particularly the NLRP3 inflammasome; and abundant acetylated α-tubulin is found to be accumulated at the perinuclear region under inducers of the NLRP3 inflammasome [17]. Acetylated α-tubulin transduces ASC to the perinuclear region on the mitochondria and assembles ASC with NLRP3 to form the entire NLRP3 inflammasome [17]. Our present study demonstrated that *P. gingivalis* infection promoted acetylation of microtubules, thereby facilitating activation of the NLRP3 and the assembly of NLRP3-ASC, which was evidenced by increased ASC speck around the nucleus.

MARK4 regulates the NLRP3 positioning and inflammasome activation mainly in a microtubule-dependent manner in macrophages [16]. MARK4 mainly regulates the microtubule dynamic by phosphorylating the microtubule-associated proteins (MAPs) and causes the detachment of MAPs from microtubules [15]. MARK4 participates in the high glucose-induced NLRP3 inflammasome activation, thus mediating IL-1β and IL-18 expression in vascular endothelial cells [23]; in addition, MARK4 deficiency inhibited hypoxia/reoxygenation (H/R)-induced NLRP3 inflammasome activation [39]; moreover, positive MARK4 expression has been observed in human atherosclerotic lesions, and MARK4 deficiency in BMDMs reduced cholesterol crystal-induced NLRP3 inflammasome activation [16]. Our present study further demonstrated that MARK4 has been implicated in bacteria-induced inflammasome activation in periodontitis. Upon inhibition of MARK4, improper positioning of NLRP3 will destroy the assembly of NLRP3 inflammasome along microtubules in the centrosome, eventually attenuating inflammasome activation [21].

The assembly of the NLRP3-ASC complex depends on cargo trafficking, which is mainly in the charge of microtubules [17,21,40]. Acetylation of α-tubulin increases the stability and polymerizations of the microtubules, enhancing the motor functions of dynein and kinesin and promoting the transport of cargo [40]. Accompanying the acetylation process of the microtubule, more ASC specks were transported as cargoes along the microtubule to the perinuclear region, whereas resveratrol (α-tubulin deacetylase) was able to reverse this process [18]. Based on our investigation, MARK4 inhibitors and MARK4 knockdown diminished the acetylation and polymerization of α-tubulin induced by *P. gingivalis*. We inferred that MARK4 might participate in the regulation of NLRP3-ASC complex assembly and eventually the pyroptosis process by acetylation/polymerizations of microtubules.

*P. gingivalis* manipulates several pathogenic factors to invade the host cells and evade immune cells, including the LPS, gingipains, and outer membrane vesicles. In the present study, we utilized live bacteria of *P. gingivalis* to infect BMDMs and mimic periodontal infection. Further studies that explore the difference of various pathogenic factors from *P. gingivalis* are needed to explore the mechanism of MARK4 activation in *P. gingivalis*-infected BMDMs. It must be noted that although OTSSP167 has high binding-affinity to the MARK4 [41], it also effectively inhibits the phosphorylation and kinase activity of maternal embryonic leucine zipper kinase (MELK) substrates and suppresses the growth of some malignancies with high expression levels of the MELK protein [42]. Moreover, the cytotoxicity of OTSSP167 and Compound 50 is still a problem, and whether such regulation of microtubule dynamics may affect bactericidal effects of macrophages need to be further investigated.

In conclusion, this study was the first to prove that MARK4 affects the NLRP3 inflammasome-dependent pyroptosis in periodontitis. Targeting MARK4 may be an effective and feasible method to combat bacterial injury and protectperiodontal tissues.

## Date available statement

All data of this study were included in the published article or are available from the corresponding author on reasonable request.
